# Structural and Functional Profiling of the Human Histone Methyltransferase SMYD3

**DOI:** 10.1371/journal.pone.0022290

**Published:** 2011-07-14

**Authors:** Kenneth W. Foreman, Mark Brown, Frances Park, Spencer Emtage, June Harriss, Chhaya Das, Li Zhu, Andy Crew, Lee Arnold, Salam Shaaban, Philip Tucker

**Affiliations:** 1 OSI Pharmaceuticals, Inc., Farmingdale, New York, United States of America; 2 University of Texas, Institute for Cellular and Molecular Biology, Austin, Texas, United States of America; 3 AltheaDx, Inc., San Diego, California, United States of America; 4 Eli Lilly and Company, San Diego, California, United States of America; 5 DiscoverElucidations, LLC. Mt. Sinai, New York, United States of America; 6 Abbott Bioresearch Center, Worcester, Massachusetts, United States of America; University of Minnesota, United States of America

## Abstract

The SET and MYND Domain (SMYD) proteins comprise a unique family of multi-domain SET histone methyltransferases that are implicated in human cancer progression. Here we report an analysis of the crystal structure of the full length human SMYD3 in a complex with an analog of the S-adenosyl methionine (SAM) methyl donor cofactor. The structure revealed an overall compact architecture in which the “split-SET” domain adopts a canonical SET domain fold and closely assembles with a Zn-binding MYND domain and a C-terminal superhelical 9 α-helical bundle similar to that observed for the mouse SMYD1 structure. Together, these structurally interlocked domains impose a highly confined binding pocket for histone substrates, suggesting a regulated mechanism for its enzymatic activity. Our mutational and biochemical analyses confirm regulatory roles of the unique structural elements both inside and outside the core SET domain and establish a previously undetected preference for trimethylation of H4K20.

## Introduction

SET domain histone methyltransferases (HMTases) mediate epigenetic post-translational histone modifications that govern transcriptional activity, in part by modulating chromatin structure and accessibility of transcription factors and RNA polymerase II (pol Il) to promoters [1]. Pol II promoters are typically repressed by histone H3 lysine 9 trimethylation (H3K9me3), H3 lysine 27 trimethylation (H3K27me3), and/or H4 lysine 20 di- and tri-methylation (H4K20me2, H4K20me3). In contrast, active pol II promoters are generally unmethylated [2], [3] and are associated with a permissive chromatin state enriched in histone H3 and H4 acetylation and H3 lysine 4 di- and tri-methylation (H3K4me2, H3K4me3) [4]. Human cancer leads to both global and gene-specific modifications of the “epigenome” [5], [6], [7], [8], [9]. Tumorigenesis is often accompanied by a general loss of repressive marks from bulk chromatin, which leads to disruption of heterochromatin structure and transcriptional repression. Alternatively, promoter-associated CpG islands can become heavily methylated during oncogenesis, resulting in local changes in chromatin structure (e.g., nucleosome repositioning) and replacement of active histone marks by repressive ones [10], [11], [12], [13], [14], [15].

**Figure 1:**
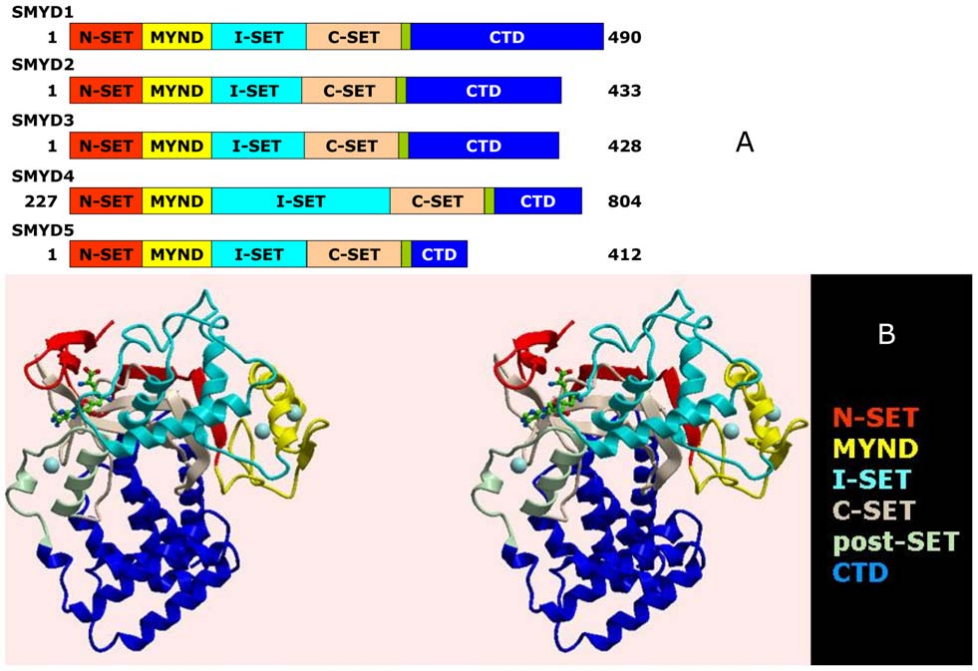
Structure of SMYD3 and its paralogs. (A) Linear representation of domain structures of SMYDs1–5. The split SET domain is shown in red (N-SET) and tan (C-SET); the MYND domain is represented in yellow and the cysteine-rich post-SET domain is displayed in pale green. Starting and ending amino acids are indicated. (B) Ribbon representation of the structure of SMYD3-Sinefungin at 1.85Å resolution in cross-eye stereo. The SET domain of SMYD3 is split into the N-SET (red) and the C-SET (tan) by an intervening MYND domain (yellow) and a Rubisco-LSMT-like I-SET region (cyan). The post-SET motif (pale green) precedes a long (∼150 residue) C-terminal domain (CTD, blue). Positions of Sinefungin (green carbons) and zinc atoms (spheres) are indicated.

SMYD3 and its 4 vertebrate paralogs (Fig. 1A) derive from an ancient family of SET HMTases with orthologs present in plants, animals, fungi, and some (typically parasitic) protozoa [16]. All SMYDs have the N-terminal terminal portion of SET (N-SET), followed by a Myeloid translocation protein 8, Nervy, and DEAF-1 (MYND) domain, N-terminal to an intermediate or linker sequence (I-SET) of variable length and configuration [17], [18]. The remainder of the SET domain (C-SET) comes next, sequentially, and includes critical catalytic folds. The SMYD SET “core” ends in a cysteine-rich zinc binding fold (post-SET). SMYDs 1–4 have an additional, previously uncharacterized ∼150 residue C-terminal domain (CTD), whereas SMYD5 has primarily insertions in its MYND and I-SET sequences. Most prototypic SET active site residues are conserved in SMYDs [19], [20], but there are notable exceptions (discussed further below). SMYD1 and SMYD3 were identified as H3K4me3-specific HMTases [5], [21], whereas SMYD2 catalyzes H3K36me2 [19]. The only previous characterization of SMYD3 HMTase was performed by Silva et al. [22], who reported that substrate release is facilitated by tumor-specific proteolysis of the SMYD3 N-terminal 34 residues. Aside from this, little has been done to establish the functional interface of SMYD3 with its substrates or its structural underpinnings.

Conversely, numerous studies have strongly implemented SMYD3 as a protooncogene in hepatocellular, colon and breast carcinoma, based on its high levels of endogenous expression, cancer-associated promoter polymorphisms, and cell proliferative effects produced by enforced SMYD3 over-expression in normal cells or SMYD silencing in tumors [5], [22], [23], [24], [25]. Approximately 80 genes have been identified as targets of SMYD3 HMTase, including Nkx2.8, a homeobox transcriptional regulator upregulated in hepatocellular malignancies as well as cell cycle mediators, oncogenes, and developmental fate determinants [5], [22], [23], [24], [25]. The considerable if not unprecedented interest in SMYD structure and its implications for putative anti-cancer drug development is evidenced by publication of three structures which appeared just prior to [26], [27] and during [28] the submission phase of this manuscript (addressed in sections below). We present here, in addition to the independent high resolution co-crystal structure of the full length human SMYD3 with the S-adenosyl methionine (SAM) analog Sinefungin, a detailed mutational and biochemical assessment of SMYD3 function. We provide a structural basis for the proposed [29], [30] differential regulation of SMYD HMTase activities via their MYND domain binding partners. We demonstrate that SMYD3 can function as a transcriptional repressor via MYND interactions as well as through hitherto undetected H4K20 HMTase activity. We show that in addition to the MYND domain, the aromatic cage structure throughout the methyltransferase active site and the unique carboxy terminal domain have the potential to regulate SMYD HMTase methylation state and substrate specificity.

## Results and Discussion

### Preferential H4K20 activity of SMYD3

**Figure 2:**
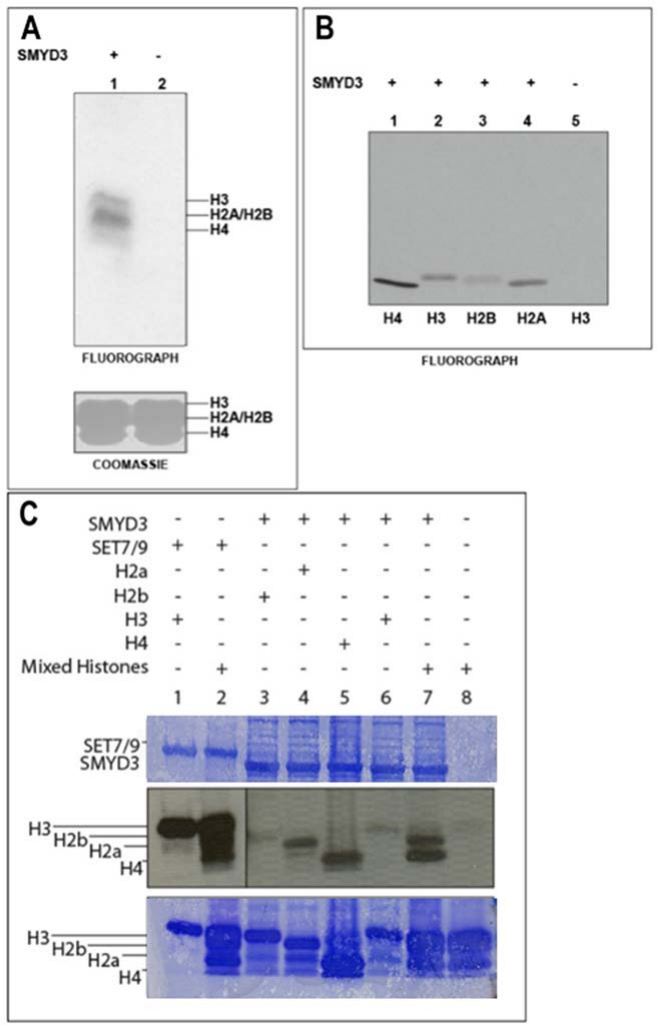
SMYD3 preferentially catalyzes histone 4 lysine methylation *in vitro*. (A, B) SMYD3 purified from baculovirus methylates all histones (H4>>H2A>H3>H2B) *in vitro.* Histone methyltransferase (HMTase) assays employed mixed histones from HeLa cells as substrate. Upper panel, fluorography showing ^3^H-incorporation into H3 (17 kD) and into species smaller bands (H2A/H2B and H4). Lower panel, Coomassie-stained SDS-PAGE gel used to verify equal loading. (C) SMYD3 purified from bacteria methylates histones H4>>H3>H2A *in vitro.* Recombinant histones or mixed histones were used, as indicated, for substrate. Fluorography is shown and the bands corresponding to each histone are indicated.

**Figure 3:**
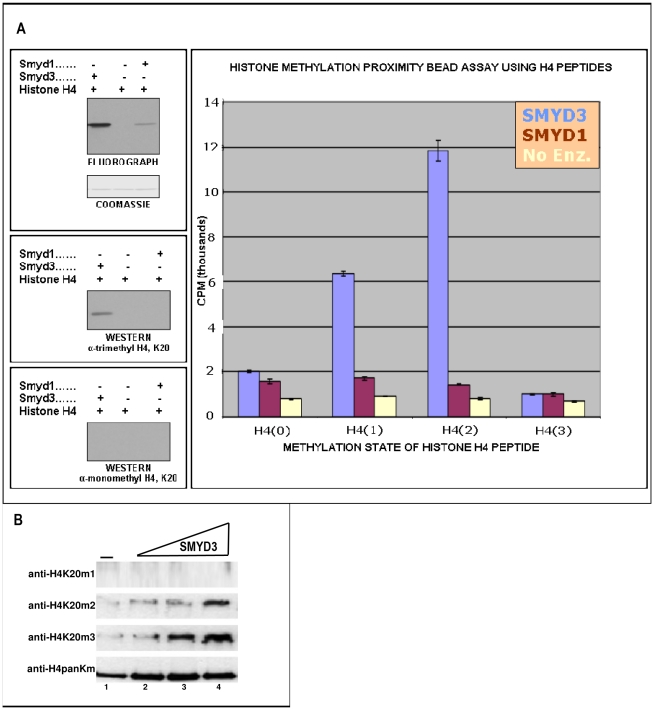
SMYD3 trimethylates H4-K20 preferentially. (A) SMYD3 trimethylates H4-K20. Right panel: unmethylated [H4(0)], mono-[H4(1)], di-[H4(2)], and, as a negative control, tri-methylated [H4(3)] peptides were employed in an *in vitro* HMTase proximity bead assay with baculoviral SMYD3 and SMYD1 (negative control). Degree of methylation was measured by scintillation counting in CPM. Left panels: Western analysis using anti-mono- and trimethyl-specific antibodies (Upstate) confirm *in vitro* specificity of SMYD3 for H4-K20me3. (B) SMYD3 preferentially trimethylates H4-K20 in reconstituted chromatin. Recombinant oocyte nucelosomes were assembled into chromatin, followed by *in vitro* HMTase assays and SDS-PAGE resolution of reaction products. SMYD3 inputs were increased from 0.5 µg to 2.4 µg, (triangle above lanes), and western analyses were performed with the indicated histone H4 methylation state-specific antibodies (middle panels), with a pan-anti-H4 (lower panel) providing a loading control for chromatin input.

Human his-tagged SMYD3 was purified following baculoviral or bacterial expression (Fig. S1). In addition to the expected H3K4me3 activity, SMYD3 methylated all histones to various degrees with highest activity for histone H4 when measured on mixed calf thymus histone acid extracts or on individual recombinant histones (Fig. 2). Western blotting with anti-H4 antibodies indicated that the maximal activity was for H4K20me3 (Fig. 3A), which was unanticipated given that this has generally been associated with establishment of heterochromatin. Using a series of synthetic H4 peptides bearing mono-, di-, and tri-methylation states at K20, we confirmed this specificity and also observed significant activity toward H4K20me2 (Fig. 3B). It is generally thought that the majority of H4K20 methylation occurs in a stepwise process in which monomethylation by the SET HMTase PR-SET7/SET8 serves as a substrate for di- and tri-methylation by SUV420H [12], [14]. That H4K20me2 served as a far better substrate than unmethylated or monomethylated species (Fig. 3B) indicated that SMYD3 alone, at least *in vitro*, is capable of progressive methylation at this lysine mark. H4K20 methylation is not a general property of SMYDs, as evidenced by the near baseline activity of SMYD1 (Fig.3A).

While it would be ideal to have a clear structural rationale for the substrate selectivity demonstrated here, crystal structures available at the time of writing do not provide enough detail to make a clear and definitive statement. Alignment of the SMYD3 (or SMYD1) structures with other structures featuring an H4 peptide fragment bound to an MTase, such as in the SET8 structure [31], shows considerable clashes between the H4 peptide and the SMYD protein. Close inspection of the overlay indicates that the H4 peptide forms part of the support for the SAM binding pocket in the SET8 structure, whereas the SAM binding pocket is fully formed and stabilized in SMYD3, independent of any substrate. Significant conformational changes would be necessary to accommodate the H4 peptide conformation as seen in [31]. Alternatively, one could use the conformation of the H3 peptide as seen in SET7/9 (discussed in more detail below), but the threading of the H4 residues onto the H3 backbone (bound to SET7/9) leads to several steric and electrostatic clashes between the modeled H4 peptide and the SMYD3 protein. Significant conformational changes and possibly water bridges would be necessary to model the H4/SMYD3 or H4/SMYD1 interaction in this peptide conformation. Further understanding of the structural roots of the observed selectivity profile requires additional studies beyond the scope of the current work.

In addition to PR-SET7 and SUV420H, only two additional SET-domain-containing proteins have been previously implicated in H4K20 methylation: The trithorax group activator Ash1 and the nuclear receptor-binding SET domain-containing protein (NSD1) (reviewed in [15]). As with SMYD3, Ash1 and NSD1 *in vitro* methylate other histone lysines in addition to K20 [32], [33]. However, whether Ash1 and NSD1 are *bona fide* H4K20 HMTases has been challenged because of the questionable specificity of the peptide antisera employed [15] and by the lack of direct confirmation both *in vitro* [34], [35], [36] and *in vivo* [11]. In support of the case of SMYD3, we observed strong di- and preferential tri-methylation of H4K20 on the most relevant *in vitro* substrate, the nucleosome (Fig. 3B). Nucleosomal H3K4me3 activity was not detected for SMYD3 (data not shown). The *in vivo* relevance of SMYD3-mediated H3K4 vs. H4K20 remains to be determined, but we return to this issue below in the context of the crystal structure.

### Conventional SET and novel features of the SMYD3-Sinefungin complex

Baculoviral SMYD3 was co-crystallized with the SAM analog Sinefungin, and the structure was solved to 1.8 Å resolution (Fig. 1B; Table 1) [37]. SMYD3-Sinefungin crystallized as 2 symmetry-related molecules/unit cell (P2_1_). However, no convincing dimer interface exists, and the mass of the purified protein following gel filtration was 50,187d (Fig. S1A), consistent with a monomeric form. Thus, we confined our analysis to the monomer of Fig. 1B.

**Table 1:** Crystallization, data collection, and refinement statistics for the SMYD3/Sinefungin structure.

Space group	P2_1_
Unit Cell	a = 58.175Å,b = 118.073Å,c = 82.901Å,α = γ = 90°,β = 91.579°
Wavelength (Å)	1.2815
Resolution (Å)	30.0–1.85
Redundancy	7.3
Unique Reflections	94,957
Completeness (%)	99.8 (99.9)^a^
<I/σ (I)>	18.4 (4.1)
Molecules/Assym. Unit	2
R/R_work_/ R_free_ b (%)	20.1/20.0/21.6

a Numbers in parentheses refer to the highest resolution shell.

b R = Σ|F_o_-F_c_|/Σ|F_o_|, where F_o_ and F_c_ are the observed and calculated structure factors, respectively. R_work_ is calculated using the formula for R, but employing only the 95% of reflections used in the refinement and R_free_ is calculated using a randomly-selected 5% subset of reflections not used in the refinement.

**Figure 4:**
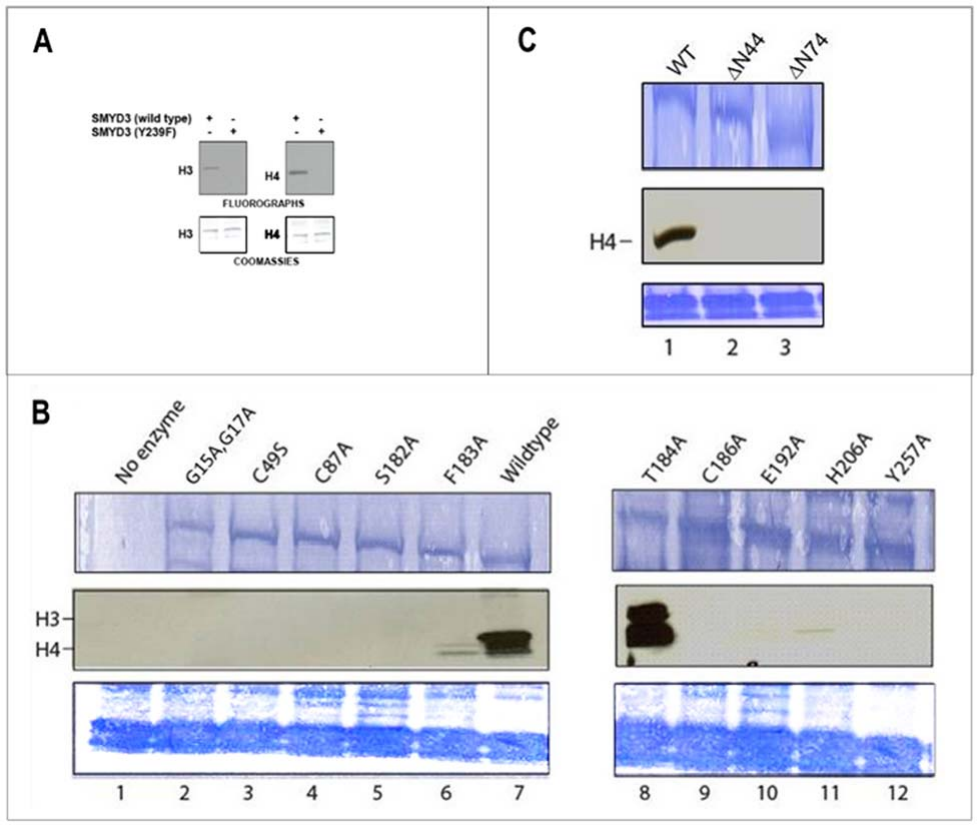
Mutational analysis of residues critical to SMYD3 structure and function. (A) Wild-type SMYD3, but not catalytic point mutant Y239F, methylates recombinant H3 and H4 in an *in vitro* HMTase assay. Upper panels: Fluorographs with bands corresponding to H3 (left) and H4 (right) indicated. Lower panels: Coomassie-stained PVDF membranes used to verify equal loading. (B) Substitution and (C) truncation mutants, constructed in E coli as described in Methods, were compared in *in vitro* HMTase assays to wildtype SMYD3 and to SET7/9. Inputs (upper panels, ∼500 ng) were assayed for ^3^H-SAM incorporation (middle panels) either on recombinant histone 4 or mixed histones, as indicated (lower panels). Alanine substitutions of most SMYD3 residues predicted to be catalytically essential eliminate HMTase activities. An exception is T184/A which, as described in the text, appears to affect H3-H4 substrate specificity (note change in relative ratios of H3/H4). N-terminal truncation through position 44 removes the entire N-SET domain, while truncation through position 74 eliminates both the N-SET domain and half the MYND domain.

The domains comprising the “core” of the split-SET (N-SET, C-SET, post-SET) of SMYD3 and the MYND domains (Fig. 1B) overlay with those of corresponding conventional domains (Figs. S2A-D) [17], [18], [38], [39], [40], [41], [42], [43], [44]. Modification of the strictly conserved and catalytically essential Y239 results in the expected loss of function (Fig. 4A). Mutation of several residues conserved within many conventional N-SETs (e.g., G15, G17) and C-SETs [17], [44] (e.g., C186, E192 and H206) abrogated SMYD3 HMTase activity (Fig. 4B), confirming the functional conservation of the split SET domain. About one third of the SMYD3 substrate binding site is formed by the Intermediate SET spacer (I-SET) region located C-terminal to MYND (Fig. 1). The significance of this variable linker region in SET substrate selectivity has already been noted [39], [41], [44], [45]. However, the SMYD3 I-SET is unusually long and exhibits extraordinary structural conservation, in lieu of primary sequence similarity, with the I-SET of the Rubisco Large Subunit Methyltransferase (RLSMT) (Fig. S2E).

**Figure 5:**
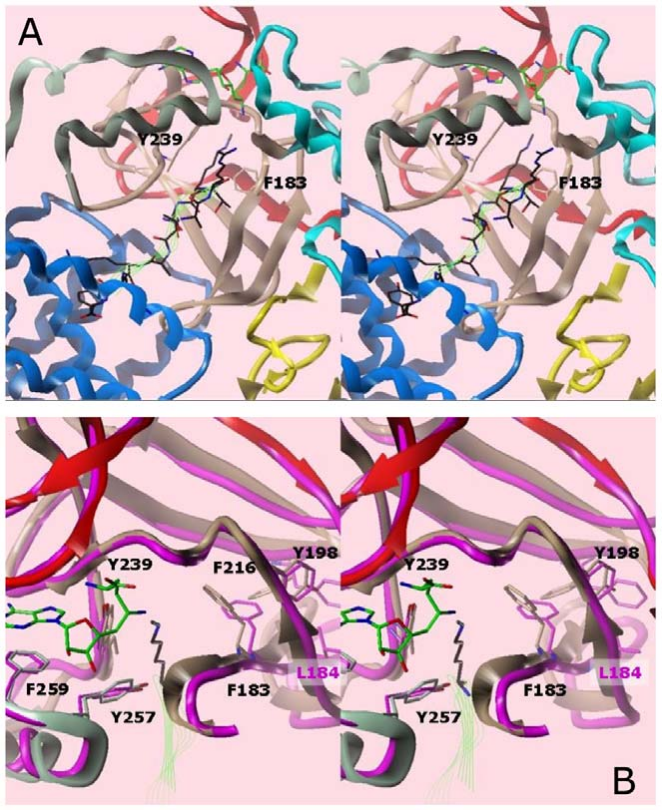
Model of the SMYD3-Sinefungin active site with the H3K4me1 peptide [**41**] in cross-eye stereo. The peptide, colored black, and with backbone traced in green ribbon, was taken from an overlay with the SET9 ternary structure. (A) Ribbon representation of the ternary complex. Substrate methyl donor and peptide are indicated as green wire bonds and ribbons, respectively. Domain colors for SMYD3 correspond to those in Fig. 1. The aromatic cage residues (Y239, F183) at the end of the lysine channel are shown explicitly. (B) Overlay of SMYD1 (magenta, PDB accession #3N71) and SMYD3 (colored by domain) proteins in the ternary model. Numbering of residues is for SMYD3.

The close structural similarity to other SET domains allowed us to superimpose onto SMYD3 the H3K4 peptide coordinates from the SET7/9 ternary complex (Fig. 5A) [41]. The peptide is bound in the conventional manner; i.e., the methyl-lysine is oriented on the opposite surface of SMYD3 from the SAM/Sinefungin methyl donor, with a narrow channel connecting the two surfaces of the SET domain. The orientation is similar to that modeled in mouse (m)SMYD1 [26] (Fig. 5B), with selectivity opportunities on either flank of the target lysine. The relatively conservative mutation T184A, which contacts the N-terminal side of the peptide, confers not only increased activity toward H4, but a striking gain of activity toward H3. The C-terminal of the modeled peptide clashes with the CTD, suggesting that the CTD also regulates the specificity of substrate binding (more below).

### The SMYD3 aromatic cage

The SMYD3 post-SET provides another commonly shared feature—an essential aromatic residue, Y257 (see Fig. 4B), that anchors against the conserved SET core to form the hydrophobic channel interface with substrate (Fig. 5B). A notable difference in SMYDs is that a critical SAM-contacting tyrosine, which occupies this position in other N-SETs (e.g.., Y335 in SET9) [17], is not conserved. To apparently compensate for the lost N-SET contact, SMYD3 and its paralogs contain a structurally homologous and essential (Fig. 4B) aromatic contact (F183 in SMYD3) within the β-1 of the C-SET (Figs. 1B and 5). The bracketing pi-cation interactions of the aromatic cage [46] are likely essential for efficient MTase activity. Extended pi cloud interactions between aromatic side-chains extending from the aromatic cage appear common in MTases. For example, residue F259 interacts with the adenine ring of Sinefungin and the Y239 of the aromatic cage, which itself packs against Y257. F216 packs against Y198 which packs against the F183 of the aromatic cage. A similar network may be seen in the SMYD1 structure, with preservation of the aromatic network around Y252, the equivalent of Y239 in SMYD3.

**Figure 6:**
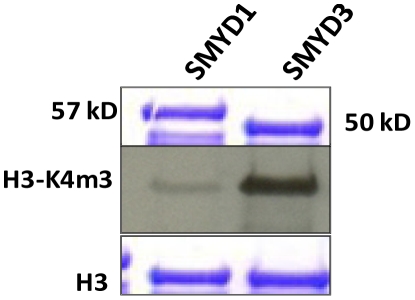
SMYD3 is more efficient than SMYD1 in trimethylation of their common substrate, H3-K4. *In vitro* assays were carried out using bacterially expressed/purified 6XHis-SMYDs and recombinant H3 (top and lower, respectively, Coomassie-stain panels). Center panel: Autoradiography of the anti-H3-K4me3 (UpState) western blot.

Despite the sequence identity and near identical backbone placement of the aromatic residues around F182 (the equivalent to F183 in SMYD3), the SMYD1 network assumes a very different set of side chain conformations, driven by the insertion of the adjacent leucine side-chain into the arrangement observed for SMYD3 (Fig. 5B). In fact, F182 in SMYD1 is rotated away from the catalytically competent conformation [26]. As suggested by this geometry, SMYD1 should be, and is, a less efficient MTase than SMYD3 in catalyzing higher methylation states of the common H3 substrate (Fig. 6). Fig S3 shows the similarities of the aromatic network among other lysine MTases, with the extent of the aromatic networking trending with the amount of methylation preferred. In general, stabilization of the biologically active conformation of the aromatic residues forming the cage surrounding the target lysine of the substrate should lead to more efficient transfer rates and therefore, indirectly, to the MTase's proclivity toward mono-, di-, or tri-methylation.

### An intact MYND domain is required for catalysis and transcriptional specificity

The MYND domain is the principal distinguishing element separating the SMYDs from other SET domain-containing proteins. MYND consists of two interlocking zinc binding folds and is present in several transcriptional regulators where it facilitates interactions with partner proteins through PXLXP motifs [47], [48], [49]. Though unfettered by SET domain constraints, the integrity of MYND is essential to SMYD3 basal function, as substitution of its Zn2+-ligating residues (C49 or C87) eliminated HMTase activity (Fig. 4B). This observation is consistent with previous analyses of the AML1/ETO MYND domain which indicated that coordination of zinc atoms is essential to maintain the intact conformation of that MYND domain, with loss of zinc coordination leading to a disordered domain [49]. Loss of coordination here also likely leads to a disordered domain, but more importantly, the lack of order affects the catalytic fidelity of SMYD3, indicating that some constraints on the linking sequence between the N- and C-SET domains exist.

**Figure 7:**
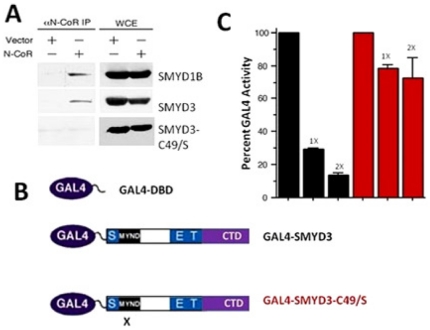
An intact SMYD3 MYND domain is required for association with N-CoR and for transcriptional repression. (A) N-CoR co-immunoprecipitates with wildtype SMYD3 but not with SMYD3 MYND domain point mutant C49/S. 293T cells were co-transfected with N-CoR, N-terminal myc-tagged SMYD3 constructs indicated, and with empty vector (vector). 48 hours post-transfection, whole cell RIPA lysates (WCL) were prepared. Fractions of the lysates were subjected to anti-N-CoR co-immunoprecipitation and the remaining 50% served as input. Western analysis was performed with anti-myc antibodies. Myc-SMYD1B, previously shown to interact with N-CoR served as a positive control. (B) Schematic of GAL4-DNA binding domain (DBD) and GAL4-fusion constructs for wild type (GAL4-SMYD3) and MYND domain-mutated (GAL4-SMYD3-C49/S) two hybrid transcription assays. X denotes the location of the C49/S mutation. (C) GAL4-SMYD3 but not GAL4-SMYD3-C49/S represses transcription of a GAL4-UAS containing luciferase reporter. 293T cells were transiently co-transfected with the 5XGAL4-SV40-luciferase reporter (1 µg) together with GAL4-DBD, or with 1 or 2 µg (indicated as 1X or 2X) of GAL4-SMYD3 (black bars) or GAL4-SMYD3-C49/S (red bars). Transfection efficiencies were normalized to co-transfected renilla luciferase, and percent GAL4 activity was determined in relation to GAL4-DBD set at 100%.

The intact MYNDs of AML-1/ETO and SMYD3 bind a common PXLXP-containing protein, the N-CoR transcriptional co-repressor (Fig. 7A) [50]. That N-CoR can bind to SMYD3 and ETO similarly is consistent with our finding that SMYD3 can act as a MYND-dependent transcriptional repressor (Fig. 7B,C). These data confirm that the nature of the MYND-bound ligand influences SMYD3 transcriptional outcomes [49].

### Potential contribution of SMYD3 and SMYD1 CTDs to catalysis

**Figure 8:**
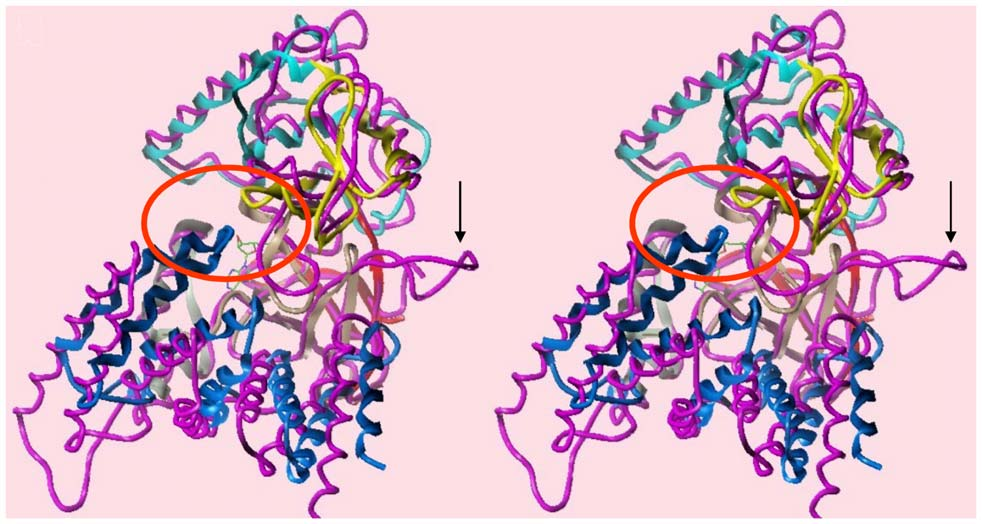
Unique carboxyl terminal domain (CTD). Comparison of the CTD orientations in SMYD1 (magenta) and SMYD3 in cross-eye stereo. The larger loop structure in SMYD1 (indicated by the arrow) forces the CTD assembly to shift such that the C-terminus no longer contacts the MYND domain, as in SMYD3. The contacts between the two domains lie within the red circle.

SMYDs1-4 have an additional ∼150 residue C-terminal domain (CTD) whose function was recently proposed [26] to regulate MTase activity of SMYDs. The SMYD3 CTD is a superhelical 9 α-helical bundle which constricts the floor of the substrate binding site opposite to the I-SET domain, preventing the trivial insertion of substrates (Fig. 8). In fact, the CTD clamps further down on the peptide binding space of SMYD3 than of SMYD1, featuring a greater superhelical pitch, such that it contacts the MYND domain (circled region of Fig. 8). The difference in pitch is likely driven by the larger turn in the C-SET domain of SMYD1 which significantly displaces the entire CTD relative to its location in SMYD3. There is still a relatively large space near the C-terminus of the modeled peptide where the inner wall of the pocket is decorated by polar residues from the CTD (mainly helix 4). We suggest that these polar residues would cooperate with the post-SET residues to select for specific sequences N- and C-terminal to the methyl-lysine, even in the absence of a significant displacement. In this context, the CTD could function as a cap necessary to bind substrates effectively and selectively.

### Complexities of SMYD3 substrate entry/release

Constrictions imposed by the I-SET, post-SET and CTD domains onto the peptide C-terminus suggest that substrate release is a complicated process for SMYD3. Silva et al. [22] reported that substrate release is facilitated by tumor-specific proteolysis of the SMYD3 N-terminal 34 residues; that is, the N-SET is "auto-inhibitory" to catalysis. To the contrary and consistent with our structure, we found that elimination of the N-SET by truncation at position 44 or 74 or by destabilizing its conserved first β-turn, eliminated HMTase activity (Fig. 4B, C). We suggest, instead, that substrate release will require a significant conformational change in the CTD, which should be readily detected by differential shifts in the geometry/contacts of unmethylated and methylated peptides.

A 1.7 Å crystal structure of the human SMYD3-sinefungin complex was reported by Sirinupong et al. [27] during the final drafting of this manuscript. Despite space group/crystal packing differences (please compare Table I of both manuscripts), the two HMTase-substrate inhibitor complexes could be virtually superimposed. Indeed, a number of active site and MYND domain residues, predicted in that paper as important for basal catalysis or PXLXP-binding interactions, were confirmed by our mutational (Fig. 4) and biochemical (Fig. 7) analyses. Based on the differential geometries adopted by the CTDs of SMYD1 and SMYD3 (*vide supra*), Sirinupong et al. [26], [27] speculated that the CTD must undergo a hinge-like movement to relieve its inherent auto-inhibition of substrate entry and/or release. However, neither of the structural analyses rules out the possibility that, at least for basal catalysis, the CTD performs a positive enzymatic function by stabilizing the active site. As shown previously [5], SMYD3 HMTase is stimulated by HSP90, a chaperone whose deregulation is also strongly implicated in a broad array of malignancies [51], [52]. It will be critical to determine if HSP90 binds directly to SMYD3, and if so, whether this interaction generates a CTD conformational change of the nature they proposed.

Another structural analysis of SMYD3 was published by Xu et al. [28] during the review process. Notwithstanding their considerably lower resolution (none better than 2.8Å), their structure overlays very closely with ours. Much as in the work of Sirinupong et al. [26], [27], Xu et al. [28] speculate on the previously observed [5], [21] association of SMYD3 with HSP90. While they do not establish a causal link, they do help establish some of the residues necessary for basal activity against an uncharacterized admixture of histones. The two residues lowering activity (D241 and D332) have a structural role, making apparently key intramolecular hydrogen bonds, while the one that does not make any intramolecular hydrogen bonds (E192) fails to alter basal activity. Interestingly, E192 is proximal to T184 in space, suggesting the trajectory of the N-termini of histones lie less towards the CTD and more towards the MYND domain, which may explain why an intact MYND domain is essential for activity. Given that Xu et al. [28] find weak but dose dependent SMYD3 HMTase activation with DNA binding to the MYND domain, one might speculate that the influence of MYND domain conformation changes may lie not only with its interactions with the C-SET residues adjacent to the catalytic binding site but also with the histone on the exterior surface.

### Conclusions

SMYD MTases share many key features in their SAM binding and lysine side-chain binding sites. A key beta-turn motif in the N-SET is essential for activity, with deletion of the motif or mutation of the superfamily signature residues G15 and G17 leading to a complete loss of activity. This motif serves as a flap that partially encloses the active site and provides residues that can interact with SAM. Targeting the disruption of this loop therefore becomes a logical objective for oncology research, as it should be sufficient to eliminate SMYD3 activity. The residues which comprise the motif are typically quite diverse and only modestly conserved, suggesting that selectivity may be achieved as well. The main drawback to targeting the loop is that the current motif features a relatively shallow groove and inhibitors would have to induce a conformational change that cannot be visualized from the current structures. Nevertheless, simulation methods could be used to explore this region of the protein.

A more likely approach to targeting SMYD3 activity is to design inhibitors that bind either the SAM- or substrate-binding pockets. Our examination of the active site suggests that disruption of the aromatic cage structure is likely to succeed, even if the site of catalysis is not occupied by an inhibitor. Differences in intramolecular aromatic-aromatic contacts lead to different stabilizations of the catalytically competent protein conformation. These differences in stability likely influence the MTase activity and hence the preference for the extent of methylation conferred on their substrates. The difference in MTase activity between SMYD1 and SMYD3 highlights this disparity: even though the sequences are identical, subtle changes in the packing influence the aromatic cages, with the more active SMYD3 retaining a stronger aromatic network than the less active SMYD1.

The MYND domain inserts into an otherwise structurally conserved SET motif that extends back to bacteria and viruses. We established that SMYD3 function is dependent on a properly folded MYND domain, suggesting that its role is not only in attracting particular binding partners but also in influencing the conformation of the N- and C-SET domains. Consistent with this hypothesis, T184 is on the far end of a beta sheet connected to the MYND domain. We establish that the rather conservative mutation of that residue to alanine leads to increased activity and promiscuity. This result suggests that small changes in the chemistry and position of the threonine side chain can lead to significant changes in catalytic activity and preference. Such changes may be possible through propagated changes in MYND domain conformation on the substrate binding pocket or may arise from changes in its direct association with a portion of the histone. More research is needed to refine these possibilities and to clarify which other residues confer the substrate preference for H4K20.

Although the MYND domain helps provide functional selectivity toward SMYD substrates, the CTD may also regulate the level of HMTase activity, serving as a cap necessary to bind substrates effectively and selectively. More experimentation is necessary to clarify the roles played by the CTD of SMYD3. New opportunities to design potent and selective agents may arise from the further characterization of these two domains and their interrelatedness to the SET domains.

Nevertheless, how do we explain the apparent biologic paradox that the oncogenic SMYD3 catalyzes histone lysine marks that promote both localized promoter activation (H3K4me3) and, even more aggressively, the repressive stabilization of heterochromatin (H4K20me3) [10], [11], [13], [53]? While global reduction of H4K20 trimethylation has been suggested to be a hallmark of human cancer [11], [15], stable and heritable H4K20-mediated repression of selected pol II genes, including tumor suppressors, has recently become appreciated as an epigenetic feature of cancer [4], [11]. For example, the tumor suppressor *target of methylation-induced silencing (TMS1/ASC)* becomes methylated and silenced in human breast and other cancers [54], [55], [56], [57]. Silencing is accompanied by a local shift from a histone activating mark, H4K16 acetylation (Ac), to H4K20 trimethylation [58]. Selective promoter-proximal "pausing" results, such that initiated Pol II accumulates just downstream of the transcription start site [59]. Taken together, SMYD3 may serve both as a repressor of tumor suppressor expression and a promoter of oncogene expression. These studies illustrate the complexities of gene-specific regulatory mechanisms in the epigenetic program and underscore the critical importance of tightly regulating the targeting of SMYD3 for regional deposition of H3K4me3 and H4K20me3.

## Materials and Methods

### Crystallography

X-ray diffraction data are summarized in Table 1. Details of protein purification and crystallization are provided below. The data were indexed and integrated using the program MOSFLM [60] and then merged using the program SCALA [61]. The subsequent conversion of intensity data to structure factor amplitudes was carried out using the program TRUNCATE [60]. The program SnB [61] was used to determine the location of Zn sites in the protein using the Bijvoet differences in data collected at the Zn peak wavelength. The refinement of the Zn sites and the calculation of the initial set of phases were carried out using the program MLPHARE [60]. The electron density map resulting from this phase set was improved by density modification using the program DM [60]. The initial protein model was built into the resulting map using the program ARP/Warp [62] and XTALVIEW/XFIT [63]; (available on request from San Diego Super Computer Center). This model was refined using the program REFMAC [60] with interactive refitting carried out using the program XTALVIEW/XFIT [63]; (available on request from San Diego Super Computer Center).

### Molecular biology

Immunoprecipitations, histone methyltransferase assays, and mutagenesis were performed as previously described [19]. Details of each of these experiments and a list of the templates and mutagenic primers employed are provided below. Dual luciferase assays using GAL4-DBD-SMYD3 wildtype and GAL4-DBD-SMYD3 mutants (C49G and C87G) were performed and normalized following transient transfection into 293T cells as previously described [19] and are detailed below.

### Cloning and baculoviral expression

The full length human SMYD3 protein (Genbank Accession No. AAH31010; SEQ. ID NO:1) was engineered to contain a C-terminal hexa-histidine tag. Sequence verified clones were each transformed into DH10 BAC chemically competent cells (Invitrogen Corporation, Cat#10361012). The transformation was then plated on selective media. 1-2 colonies were picked into minipreps and bacmid DNA isolated. The bacmids were transfected and expressed in *Spotoptera frugiperda* (SF9) cells using the following standard Bac to Bac protocol (Invitrogen Corporation, Cat.#10359-016) to generate viruses for protein expression. SF9 cells were used for 48 hr expressions in SF-900 II media.

The full length cDNA of HSP90 was cloned from Hep G2 cells [ATCC HB-8065]. The chaperone HSP90 was co-expressed with SMYD3 by co-infection with virus for each. Cells were collected by centrifugation and frozen pellets were used for purification of full length SMYD3. These procedures resulted in expression of SMYD3 and HSP90 with 3 amino acids added to their N-terminal end (MAL) and an additional 8 amino acids (EGHHHHH) added to the C-terminal end of SMYD3.

### Mutagenesis, cloning, and bacterial expression

Point mutants were generated using the GeneEditor *in vitro* Site-Directed Mutagenesis System (Promega) according to the instructions of the manufacturer For PCR, samples were heated to 94°C for 5 min, subjected to amplification for 16 cycles of 0.5 min at 94°C, 0.5 min at 55°C, and 0.5 min at 68°C and extended after the last cycle at 72°C for 7 min. Polyhistidine (6xHis)-tagged SMYD3 wildtype, truncation and substitution mutants were cloned into Gateway (Invitrogen) pET™-DEST42. High level expression was induced by IPTG in *E. coli* strains MG232 (Scarab LTM) or Hsp90Plus^TM^ (Expression Technologies Inc). Primers and mutagenic oligos were:

### Substitution mutants

G15,17A

cgccaacag ggg aaa ggg ctgcgcgccgtg          + strand

cgccaacag gcg aaa gcg ctgcgcgccgtg         forward

CACGGC GCGCAGC GCGTTTGCCCTGTTGGCG reverse

C49S

cgtggcgtcg tcg cgaccgctgcctt          + strand

cgtggcgtcg cgc cgaccgctgcctt          forward

AAGGCAGCGGTCGGCGACGACGCCACG reverse

C87A

cacaagcgggaa tgc aaatgccttaaa        + strand

cacaagcgggaa gcc aaatgccttaaa        forward

TTTAAGGCATTTGGCTTCCCGCTTGTG reverse

S182A

ctgatctgcaac tct ttcaccatctgt          +strand

ctgatctgcaac gct ttcaccatctgt         forward

ACAGATGGTGAAAGCGTTGCAGATCAC reverse

F183A

ttctgcaactct ttc accatctgtaat          +strand

atctgcaactct gcc accatctgtaat         forward

ATTACAGATGGTGGCAGAGTTGCAGAT reverse

T184A

tgcaactctttc acc atctgtaatgcg         +strand

tgcaactctttc gcc atctgtaatgcg         forward

CGCATTACAGATGGCGAAAGAGTTGCA reverse

C186A

tctttcaccatc tgt aatgcggagatg         +strand

tctttcaccatc gct aatgcggagatg         forward

CATCTCCGCATTAGCGATGGTGAAAGA reverse

E192A

gcggagatgcag gaa gttggtgttggc         +strand

gcggagatgcag gca gttggtgttggc         forward

GCCAACACCAACTGCCTGCATCTCCGC reverse

H206A

ctttgctcaat cac agctgtgacccc          +strand

ctttgctcaat gcc agctgtgacccc          forward

GGGGTCACAGCTGGCATTGAGCAAAGA reverse

Y257A

ctgagggaccag tac tgctttgaatgt            +strand

ctgagggaccagg cct gctttgaatgt            forward

CACATTCAAAGCAGGCCTGGTCCCTCAGCTA reverse

N-terminal truncations.

d44

gaattcCGTGGCGTCGTCTGCGACCGC  forward

gaattcCATGGTGCCTGCTTTTTTGTACA reverse

d74

gaattcCAGAAAAAAGCTTGGCCAGA    forward

gaattcCATGGTGCCTGCTTTTTTGTACA reverse

### Protein purification

Frozen cells were lysed in buffer [50 mM Tris-HCl pH 7.7, 250 mM NaCl with protease inhibitor cocktail (Roche Applied Science, Cat. #11-873-580-001)] and centrifuged to remove cell debris. The soluble fraction was purified over an IMAC column charged with nickel (GE Healthcare, NJ), and eluted under native conditions with a step gradient of 10 mM, then 500 mM imidazole. Proteins were then further purified by gel filtration using a Superdex 200 column (GE Healthcare, NJ), into 25 mM Tris-HCl pH 7.6, 150 mM NaCl, and 1 mM TCEP. Protein was pooled based on SDS-PAGE and concentrated to 1-10 mg/ml.

### Crystal preparation

Diffraction quality crystals were obtained by hanging or sitting drop containing 0.75 µl of protein 10 mg/ml and 1 mM Sinefungin in 25 mM TrisHCl pH 7.6, 150 mM NaCl, 1 mM TCEP and 0.75 µL reservoir solution: 100 mM Tris-HCl pH 8.5, 17% PEG 20 K, 100 mM Magnesium Chloride hexahydrate in a sealed container containing 500 µL reservoir solution, incubated overnight at 21°C. Crystals were also grown with a reservoir solution of 100 mM HEPES pH 7.5, 16% PEG 3350, 200 mM Magnesium Chloride.

The crystals were individually harvested from their trays and transferred to a cryoprotectant consisting of 75–80% reservoir solution plus 20–25% glycerol or PEG400. After ∼2 min, crystals were collected and transferred into liquid nitrogen and then transferred to the Advanced Photon Source (Argonne National Laboratory), where a two wavelength MAD experiment was collected, using a Zn peak wavelength and a high energy remote wavelength.

### Immunoprecipitation (IP) and Western blotting

293T cells were transiently transfected, harvested 48 hours later, and then lysed in RIPA buffer (150 mM NaCl, 1% NP-40, 0.5% DOC, 50 mM Tris pH 8, 0.1% SDS) containing protease inhibitors (Roche Molecular Biochemicals, Indianapolis, IN). Cell supernatants were incubated with primary anti-tag mAb or polyclonal anti-H3 Ab (0.5–2 ug/ml) centrifuged at 4°C, and then incubated with protein A-Sepharose/protein G PLUS-agarose (Santa Cruz Biotechnology) at 4°C with rotation for 1 hour. Resulting immune complexes were washed 6 times and immunoprecipitated proteins were resolved on 8–15% SDS-PAGE. Separated proteins were transferred to nitrocellulose (Protran BA, Schleicher and Schuell, NH), blocked using 5% nonfat milk (10 g nonfat milk, 150 mM NaCl, 10 mM Tris pH 8, 0.05% Tween-20) overnight at 4°C. Membranes were incubated with 1° antibody for 1 hour at room temperature, extensively washed, then incubated with 2° antibodies for 1 hour at room temperature. Blots were exposed and developed using the ECL blot detection reagent (Amersham Pharmacia Biotech) according to the instructions of the manufacturer.

### Histone methyltransferase assays

For *in vitro* HMTase assays, SMYD3 proteins (0.1–1 µg) +/− equivalent amt. of human HSP90α (Assay Designs, Ann Arbor, MI, USA, cat. no SPP-776D) were incubated with 1 µg of mixed histones from calf thymus (Sigma) or recombinant core histones (Upstate), or with 1 µg reconstituted chromatin generated from oocyte nucleosomes (graciously provided by Dr. Yali Dou, Univ. Michigan Med School) prepared as described previously [64], [65]. Recombinant oocyte histones were assembled onto a 201 bp ‘601’ DNA template [66] by mixing ∼1.5 µg octamers with 1 µg DNA template in a volume of 10 µl containing 2 M NaCl, 10 mM Tris (pH 8.0), 0.1 mM EDTA, and 10 mM β-mercaptoethanol), followed by stepwise, 10-fold reduction of salt by addition of Tris–EDTA to a final concentration of ∼0.2 pmol nucleosome/µl 200 mM NaCl/Tris–EDTA). For radioactive based assays, 2 µCi S-adenosyl-L–[methyl-^3^H] methionine (SAM; Amersham Biosciences) was included as a methyl donor. All reactions were carried out in 40 µl HMT reaction buffer (10 mM dithiothreitol, 100 mM NaCl, 4 mM MgCl2, and 50 mM Tris-HCl at pH 8.8) at 30°C for 3 hours. An 18% SDS-PAGE gel was used to resolve the samples and fluorography was used visualize positive methylation. Substrate loading was visualized by Coomassie blue staining.

Specificity of SMYD3 activity was determined, following transient transfection into 293T cells, by incubating immunoprecipitated proteins with recombinant histones and 20 µM unlabelled SAM (Sigma) in 40 µl HMT reaction buffer at 30°C for 1 hour. Western blot analysis was conducted using antibodies against H3K4me2, H3K4me3, H3K9me2, H3K9me3, H3K27me3, H3K36me2, H3K79me2, H4K20me2, and H4K20me3 (all from Upstate, Charlottesville, VA).

Preferential H4 methylation state catalyzed by SMYD3 was confirmed by proximity bead HMT assays as follows: 2 µCi of ^3^H-SAM (Amersham Biosciences) were incubated with 0.1 µg of SMYD3 and 0.1 µg of histone H4 peptide, non-, mono-, di-, or tri-methylated at K20 (sequence of the peptide: acetyl-GGKGLGKGGAKRHRKVL-biotin). The assay was carried out for three hours at 30°C in 20 µl HMT reaction buffer. At the end of the incubation time, 100 µl of binding buffer (1x PBS containing 1% NP-40 and 0.1% SDS) was added. The substrate was then precipitated using 10 µl of Streptavidin PVT SPA Scintillation Beads (Amersham Biosciences; used as 50% slurry in binding buffer) for one hour at room temperature on a rocking platform, followed by five washes in binding buffer and scintillation counting.

### Transcription assays

The SV40-luciferase reporter, containing five copies of the GAL4-UAS, was obtained from J. Milbrandt [19]. pRL-TK was purchased from Promega. The GAL4-SMYD3 WT and MYND mutant mammalian expression vectors were constructed by PCR amplification (5′ ATG CGC GCC GAG GCC CGC; 3′ TCA GTG GCT CTC AAT CTC CTG) and restriction digestion (Not I; Xba I) followed by subcloning into the GAL4-DBD plasmid [20]. Dual luciferase assays were performed and normalized following transient transfection into 293T cells as previously described [19].

## Supporting Information

Figure S1 **Expression and purification of recombinant human SMYD3.** (A) Baculoviral SMYD3. 6X-his-SMYD3 was expressed in sf9 cells as detailed in Methods, purified by Ni-NTA, HiTrap-Q, and Superdex-75 column chromatography (left) and confirmed for purity by mass spectrometry (right). SMYD3 purified as a monomer of predicted (50189) mass. These fractions were suitable for crystallization and further biochemical analyses (described in text). (B) Bacterial SMYD3. 6X-his-SMYD3 (wildtype, catalytic mutant H206/A, and other mutants analyzed in Suppl. Fig. 4) were cloned into Invitrogen Gateway plasmids as described in Methods. Following IPTG-induction in Scarab MG232 (left), proteins were purified by Ni-NTA (center) and confirmed with polyclonal anti-SMYD3 (right).
(TIF)

Figure S2 **Conventional SET and MYND domain architectures are unaltered in SMYD3.** (A–C) Ribbon representations in cross-eye stereo of the N-SET(A), C-SET (B), and POST-SET (C) domains of SMYD3 (colored by domain as in Fig. 1) superimposed on the corresponding regions of SMYD1 (magenta), SET8 (red brown), SET9 (black), Rubisco LSMT (white), Dim5 (green), Clr4 (blue-green), and the viral SET of the Chlorella virus (gold). Zn locations are indicated by spheres. Sinefungin is represented in green wireframe. (D) Structure of the MYND domain in cross-eye stereo of SMYD3 (yellow) superimposed on the MYND domains of ZMYND10 (green), ETO (white), CBFA2TI (black), and SMYD1 (magenta). Zn locations are indicated by spheres. (E) Overlay of the complete SET domain of SMYD3 (colored by domain as in Fig. 1) with those of SMYD1 (magenta) and Rubisco LSMT (white) in cross-eye stereo. Sinefungin is represented in green wireframe. Of all MTase structures currently available, only these three almost completely overlay, including the commonly conformationally and sequentially variable I-SET region.
(TIF)

Figure S3 **Aromatic residues in the catalytic site in cross-eye stereo.** Comparison of the SMYD3 catalytic site (colored by domain) with the corresponding site in (A) SET8 (red brown), (B) SET7/9 (black), (C) Rubisco LMST (white), (D) DIM5 (green), (E) CLR4 (blue green), and (F) the SET domain from the Chlorella virus (gold). The modeled lysine from the SET7/9 structure (black carbons) and Sinefungin (green carbons) are displayed for reference.
(TIF)
