# Supplementary figures and images for: Structural and Functional Profiling of the Human Histone Methyltransferase SMYD3
Source: PLoS One. 2011 Jul 14;6(7):e22290. doi: 10.1371/journal.pone.0022290 (PMC3136521; doi:10.1371/journal.pone.0022290)

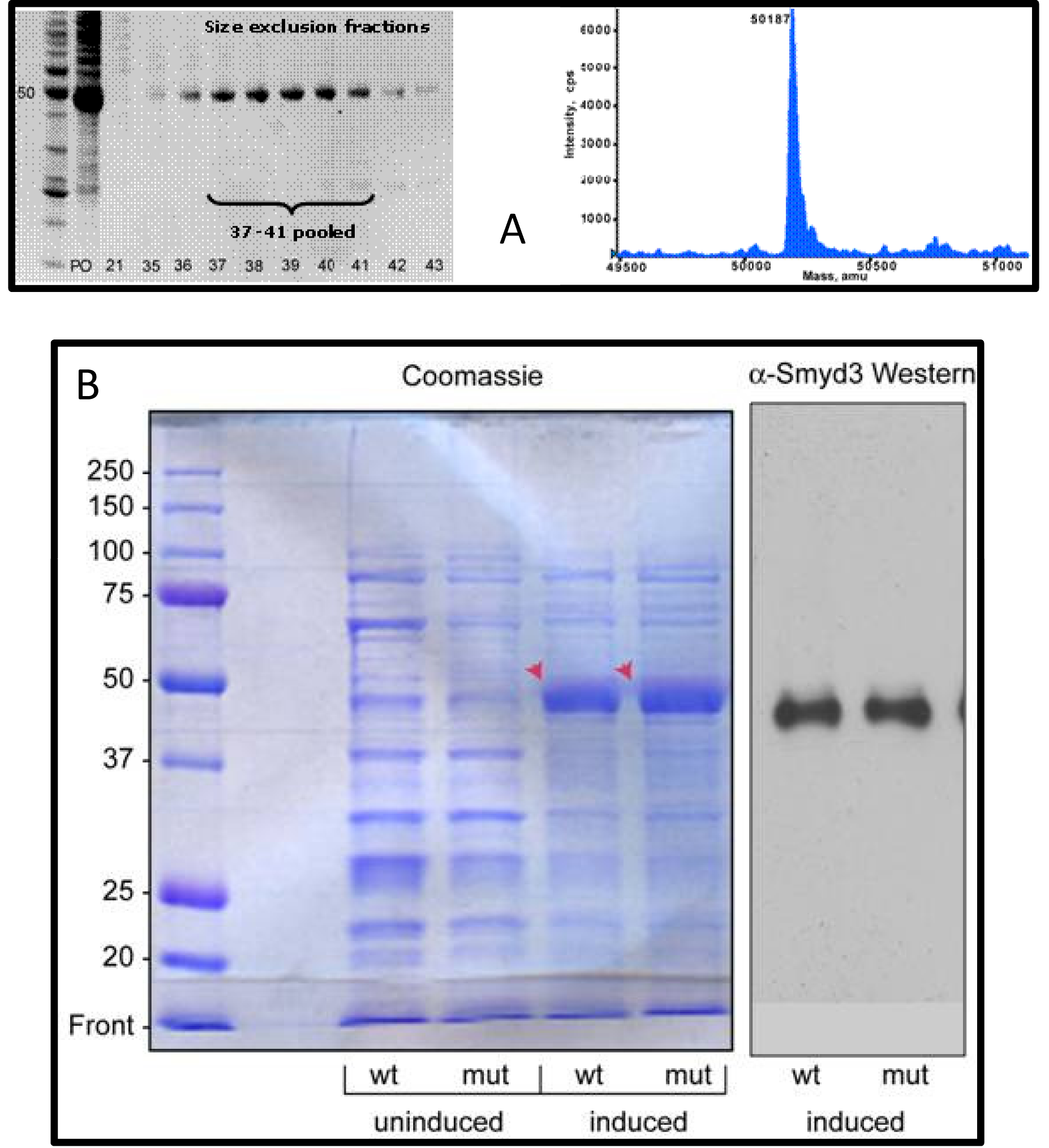

Supplement: Figure S1 — Expression and purification of recombinant human SMYD3. (A) Baculoviral SMYD3. 6X-his-SMYD3 was expressed in sf9 cells as detailed in Methods, purified by Ni-NTA, HiTrap-Q, and Superdex-75 column chromatography (left) and confirmed for purity by mass spectrometry (right). SMYD3 purified as a monomer of predicted (50189) mass. These fractions were suitable for crystallization and further biochemical analyses (described in text). (B) Bacterial SMYD3. 6X-his-SMYD3 (wildtype, catalytic mutant H206/A, and other mutants analyzed in Suppl. Fig. 4) were cloned into Invitrogen Gateway plasmids as described in Methods. Following IPTG-induction in Scarab MG232 (left), proteins were purified by Ni-NTA (center) and confirmed with polyclonal anti-SMYD3 (right). (TIF) [file pone.0022290.s001.tif]

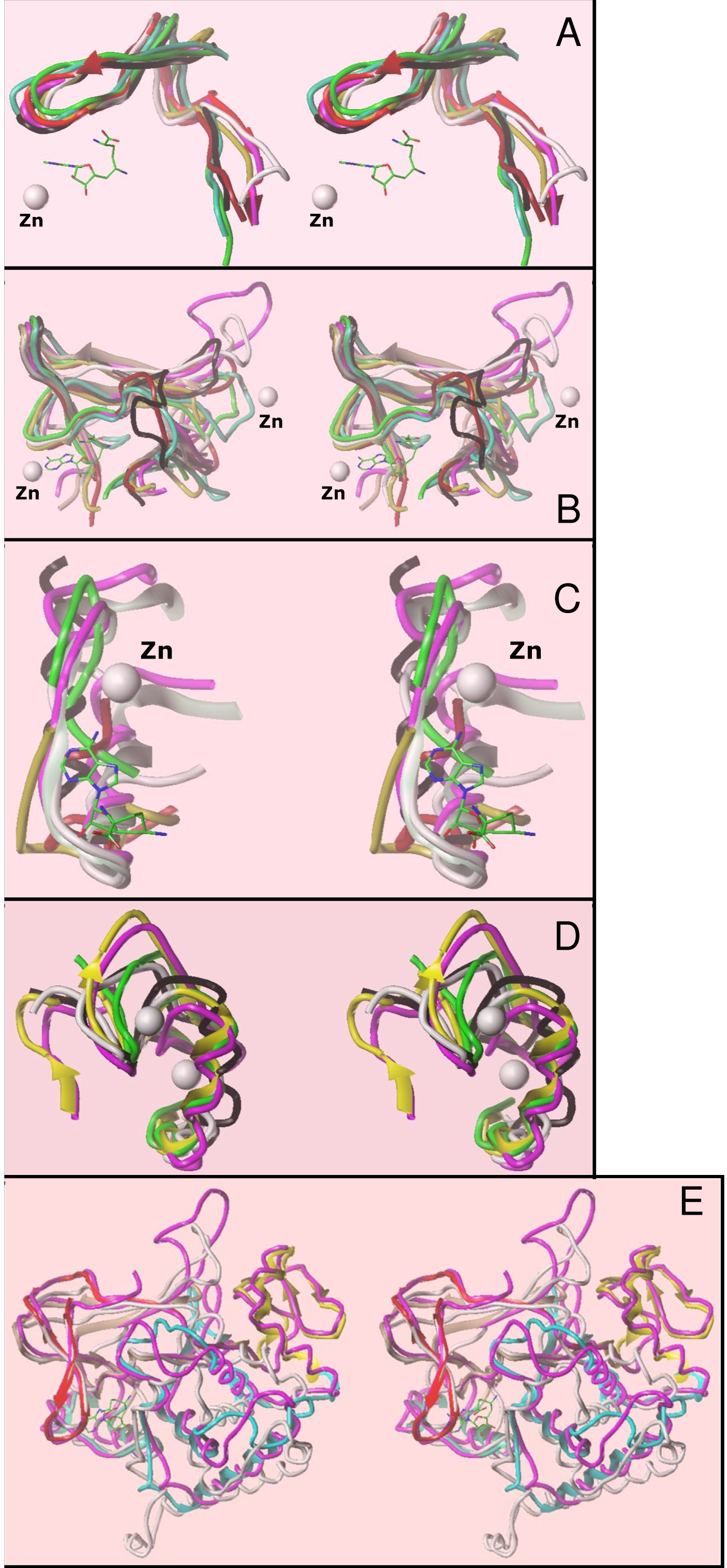

Supplement: Figure S2 — Conventional SET and MYND domain architectures are unaltered in SMYD3. (A–C) Ribbon representations in cross-eye stereo of the N-SET(A), C-SET (B), and POST-SET (C) domains of SMYD3 (colored by domain as in Fig. 1) superimposed on the corresponding regions of SMYD1 (magenta), SET8 (red brown), SET9 (black), Rubisco LSMT (white), Dim5 (green), Clr4 (blue-green), and the viral SET of the Chlorella virus (gold). Zn locations are indicated by spheres. Sinefungin is represented in green wireframe. (D) Structure of the MYND domain in cross-eye stereo of SMYD3 (yellow) superimposed on the MYND domains of ZMYND10 (green), ETO (white), CBFA2TI (black), and SMYD1 (magenta). Zn locations are indicated by spheres. (E) Overlay of the complete SET domain of SMYD3 (colored by domain as in Fig. 1) with those of SMYD1 (magenta) and Rubisco LSMT (white) in cross-eye stereo. Sinefungin is represented in green wireframe. Of all MTase structures currently available, only these three almost completely overlay, including the commonly conformationally and sequentially variable I-SET region. (TIF) [file pone.0022290.s002.tif]

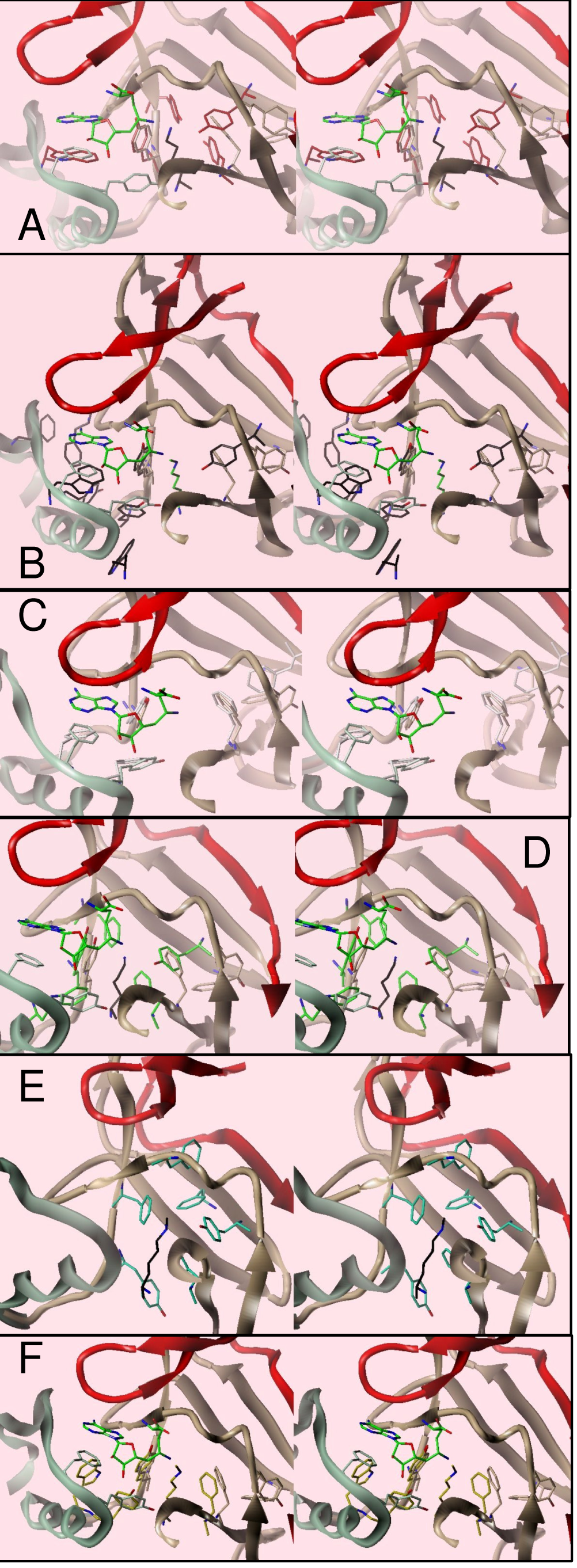

Supplement: Figure S3 — Aromatic residues in the catalytic site in cross-eye stereo. Comparison of the SMYD3 catalytic site (colored by domain) with the corresponding site in (A) SET8 (red brown), (B) SET7/9 (black), (C) Rubisco LMST (white), (D) DIM5 (green), (E) CLR4 (blue green), and (F) the SET domain from the Chlorella virus (gold). The modeled lysine from the SET7/9 structure (black carbons) and Sinefungin (green carbons) are displayed for reference. (TIF) [file pone.0022290.s003.tif]
